# Quantitative Measurements for Factors Influencing Implementation in School Settings: Protocol for A Systematic Review and A Psychometric and Pragmatic Analysis

**DOI:** 10.3390/ijerph191912726

**Published:** 2022-10-05

**Authors:** Sara Hoy, Björg Helgadóttir, Åsa Norman

**Affiliations:** 1 Department of Movement, Culture, and Society, The Swedish School of Sport and Health Sciences (GIH), 114 86 Stockholm, Sweden; 2 Department of Physical Activity and Health, The Swedish School of Sport and Health Sciences (GIH), 114 33 Stockholm, Sweden; 3 Department of Clinical Neuroscience, Karolinska Institute, Tomtebodavägen 18A, 171 77 Stockholm, Sweden; 4 Department of Psychology, Stockholm University, 106 91 Stockholm, Sweden

**Keywords:** systematic review, implementation, dissemination, measurement, barriers, facilitators, schools, psychometrics, validity, reliability

## Abstract

Introduction: In order to address the effectiveness and sustainability of school-based interventions, there is a need to consider the factors affecting implementation success. The rapidly growing field of implementation-focused research is struggling to determine how to assess and measure implementation-relevant constructs. Earlier research has identified the need for strong psychometric and pragmatic measures. The aims of this review are therefore to (i) systematically review the literature to identify measurements of the factors influencing implementations which have been developed or adapted in school settings, (ii) describe each measurement’s psychometric and pragmatic properties, (iii) describe the alignment between each measurement and the corresponding domain and/or construct of the Consolidated Framework for Implementation Research (CFIR). Methods: Six databases (Medline, ERIC, PsycInfo, Cinahl, Embase, and Web of Science) will be searched for peer-reviewed articles reporting on school settings, published from the year 2000. The identified measurements will be mapped against the CFIR, and analyzed for their psychometric and pragmatic properties. Discussion: By identifying measurements that are psychometrically and pragmatically impactful in the field, this review will contribute to the identification of feasible, effective, and sustainable implementation strategies for future research in school settings.

## 1. Introduction

To date, a smorgasbord of interventions have been designed across disciplines to affect different outcomes in educational settings, yet these highly supported programs often yield varied effects and sustainability due to a poor understanding of their implementation [1,2]. Dissemination and implementation (D&I) science has been on the rise, especially during the last two decades. This research can be described as the study of the integration of interventions (individual or collective practices, policies, or programs) into real-world settings [3,4]. One real-world setting in which youth spend approximately a third of their time is school. School settings are important contexts that influence the social, intellectual, and health development of children and adolescents [5]. Studies in school settings have consistently demonstrated that interventions are rarely implemented as designed, and that contextual factors affecting implementation need to be further studied and addressed at multiple levels, from the individual level to the policy level [6]. For example, Saunders and colleagues [7] found that there was a relationship between the level of implementation and successful program outcomes in their school-based intervention designed to promote physical activity in high-school girls. Girls in high-implementing schools reported engaging in vigorous physical activity to a greater percentage; moreover, high-implementing schools also significantly differed in administrator-reported organizational-level components compared to other schools [7]. Despite early attempts by Berman and McLaughlin [8] to question the assumption that innovations were implemented in school settings as intended, much seems to remain unaddressed in the design and evaluation within school-intervention research to ensure the effectiveness and sustainability of implementation [1,6]. The lack of research on the barriers and facilitators impacting implementation is highlighted among reviews on school-based interventions across different outcomes, such as physical activity [9,10,11], tobacco or substance use [12], mental health promotion [13], and technology use among teachers in education [14], to mention a few.

Several frameworks have been developed to conceptualize and capture our understanding of how interventions are ‘woven together’ with a certain setting. When Durlak and DuPre [15] compiled qualitative and quantitative data on the factors affecting the implementation process in their review, they found strong support for 23 factors divided over five categories. The ecological framework they presented based on their results shows the vast complexity of factors influencing implementation success. These factors span from the community level, to the characteristics of the innovation and the provider, to organizational capacity and support systems, to specific practices, processes, and staffing considerations [15]. Several frameworks within implementation science address similar factors, where one of the most widely used across a wide range of studies is the Consolidated Framework for Implementation Research (CFIR) [16,17,18]. The CFIR consists of 39 constructs divided over five domains; outer setting, inner setting, intervention characteristics, characteristics of individuals, and process. It is based on a spectrum of construct terminology and definitions compiled into one structured framework [19], and was designed to label and define constructs to describe contextual factors [20]. The framework especially focuses on barriers and facilitators at multiple levels that may impact implementation success, similar to the ecological framework by Durlak and DuPre [15].

To study the effectiveness and sustainability of school-based interventions, there is a need to consider the factors affecting implementation success. However, this rapidly growing field of D&I-focused research is struggling with how to assess and measure relevant constructs [21,22,23]. There are difficulties concerning the understanding of which variables, when, and at what level to assess, as well as the synonym, homonym, and instability of constructs, to name a few [24,25]. As an example, Weiner and colleagues [26] discuss the implementation climate as an important factor to consider for implementation effectiveness. However, there are no standard measurements for assessing implementation climate, there are inconsistencies in how this construct is defined, few instruments have been used more than once, and these instruments are rarely assessed for reliability and validity [26]. Several other studies have reported findings based on instruments that do not have established psychometric properties addressing their validity and reliability. For instance, the construct of organizational learning is highlighted by Bowen and colleagues [27] in the context of interventions to improve student achievement. The authors mention obstacles for designing and evaluating interventions to promote the operation of schools as learning organizations, mainly due to few available assessment tools with established reliability and validity [27]. These issues further limit the interpretability, comparability, and transferability of these studies [28]. Previous research has identified the need for not only strong psychometric measures but also pragmatic measures assessing their likelihood of being used [29]. Key criteria should be further considered in the design of pragmatic measures, such as the importance to stakeholders and researchers, a low burden to assessors and respondents, and being actionable [29].

The Society for Implementation Research Collaboration (SIRC) and their Instrument Review Project [24,30] is a clear example of current work within the field. To our knowledge, multiple previous reviews [22,24,31,32,33,34,35,36] have been performed to map out implementation measurements in public health and community settings, beyond health-care and clinical settings where these types of studies are most commonly performed. This review contributes to expanding previous knowledge by assessing measurements limited to school settings, as well as by capturing the most recent work in an area of research that is rapidly evolving. Therefore, the aims of this review are to (i) systematically review the literature to identify measurements of the factors influencing implementation which have been developed or adapted in school settings, (ii) describe each measurement’s psychometric and pragmatic properties, and (iii) describe the alignment between each measurement and the corresponding domain and/or construct of the Consolidated Framework for Implementation Research (CFIR).

## 2. Materials and Methods

### 2.1. Design and Guiding Frameworks

The current review uses systematic review procedures, and reports in accordance with the updated Preferred Reporting Items for Systematic Reviews and Meta-Analyses (PRISMA 2020) guidelines with its extension for searches (PRISMA-S) [37,38]. The extension for protocols (PRISMA-P) for this review can be found in Additional File 1.

All the included measurements will be assessed based on the recommended best practice for psychometric properties and pre-defined criteria. Further, the included measurements will be mapped against the domains and constructs of the Consolidated Framework for Implementation Research (CFIR) [19]. The CFIR is one of the most widely utilized and acknowledged meta-frameworks within D&I [16,17], and captures the factors influencing implementation success.

This review has been prospectively registered at the Open Science Framework [39] at osf.io/fxhmv.

### 2.2. Eligibility Criteria

All the publications included will be peer-reviewed journal articles containing original research, in English and several Nordic languages (Swedish, Norwegian, Danish, and Icelandic), and published after the year 2000. This date restriction is chosen mainly because published work within D&I has been on the rise during the last two decades. Additionally, the included publications must report research from school settings: including primary and secondary school, and excluding preschool, tertiary, and vocational education. This is mainly due to the fact that the included settings are similar enough concerning organization and contextual environments. Research populations could involve school stakeholders’ such as students, teachers, school leaders and management, school nurses, psychologists, assistants, and educators or similar school staff. Only quantitative measures will be included, whereas, qualitative methods will be excluded. The eligibility criteria are summarized in Table 1.

Abstracts describing editorials, commentaries, conference abstracts, dissertations, and other grey literature, as well as publications which reported on measures developed using exclusively qualitative methods were judged ineligible.

### 2.3. Search Strategy

A detailed search strategy will be developed by researchers (S.H., Å.N.) together with the Karolinska Institute University Library (KIB) search specialist staff. KIB will further be enlisted to perform the searches.

To identify related measurements, we will systematically search the following six electronic databases: (i) Medline (Ovid); (ii) ERIC (ProQuest); (iii) PsycInfo (Ovid); (iv) Cinahl (EBSCO); (v) Embase (embase.com), and (vi) the Web of Science Core Collection (Clarivate). Consistent with our aim to identify and assess implementation-related measurements in the educational-, behavioral-, and health-spaces, the search string will be built on three core levels: (a) terms for implementation, (b) terms for measurement, and (c) terms for school settings. To address potential terminological inconsistencies within the multiple fields covered in the current review, we will consult experts on search terms within educational, implementation, and public health research. A test strategy for the search terms will be conducted in Web of Science Core Collection (Clarivate), then reviewed by the research team and KIB together. The final strategy will be adapted to fit the five remaining databases.

While this review is mainly situated within public health and implementation science, we will also address articles within educational science as a result of our chosen setting being schools. Some of the commonly used search categories from the PICO-framework [40] of systematic reviews are sometimes less useful within other types of non- medical reviews, such as educational reviews, where the outcomes included might range, control groups are not always present, and studies without an intervention are important to include [41]. This issue will be addressed through searching both generic and subject specific bibliographic databases (e.g., ERIC), hand-searches, contact with experts, and citation checking [42]. These additional manual searches will be performed throughout the search period, and reference lists of earlier reviews will be screened for additional eligible articles (S.H.).

### 2.4. Identification of Eligible Publications

Duplicate abstracts will initially be removed by KIB [43]. All remaining records will be screened in two stages. First, the title and abstract will be screened independently by two researchers (S.H., B.H.) in accordance with the inclusion and exclusion criteria, where obviously irrelevant studies will be removed. This initial step will be performed using Rayyan software [44], where the independent screening of the title and abstract will be carried out blinded between researchers. Additionally, 10% of the initial screening of abstracts will be cross-checked by a third researcher (Å.N.). When all records are initially screened, a comparison between the screenings will be made. In the case of discrepancies, all three researchers (S.H., B.H., Å.N.) will discuss the issue until a consensus has been reached. Second, full-text versions of publications will be obtained for the included abstracts to be screened in further detail by two researchers (S.H., B.H.), also independently. Again, a 10% cross-checking of the full texts will be carried out by a third researcher (Å.N.). If a decision cannot be made regarding a full-text article’s eligibility, all three researchers will discuss the issue until a consensus has been reached. A detailed description of the screening process will be presented as text and with a figure of the PRISMA flow-chart of the study selection.

Once measurements are mapped to CFIR’s domains and psychometric and pragmatic criteria, we will perform the analysis of each measurement. The final set of publications included will be carefully evaluated for the presence of CFIR-related constructs and items, and their psychometric and pragmatic properties.

### 2.5. Extraction of Data from Eligible Publications

Data will be extracted and compiled through a systematic process in accordance with a project codebook which covers the study characteristics, psychometric and pragmatic properties, and CFIR domains, as developed by three researchers (S.H., B.H., Å.N.).. For the purpose of a similar pre-understanding of the topic, the team will read articles on related measure evaluation systems (e.g., COSMIN [45]) and psychometric properties [28,29,46,47,48], and implementation science reviews [22,24,31], as well as the original work of the theoretical framework CFIR [19]. As a first stage, the team will extract data according to the project codebook from two included articles, independently of one another. The similarities and differences in coding will be addressed and discussed until a consensus has been reached, to initially obtain an as comparable data extraction and coding process as possible.

### 2.6. Study Characteristics

The characteristics of each study will be extracted, synthesized, and reported, such as the country, setting, sample and study population, characteristics of the innovation being assessed, and guiding theoretical frameworks.

### 2.7. CFIR Coding

The factors influencing the implementation being assessed in each measurement will be coded according to CFIR’s 5 domains and 39 constructs using a developed project codebook, based on the original work of Damschroder and colleagues [19] and data analysis tools available online [49]. The coding process will focus on assessing the items, constructs, and domains of each instrument and evaluating how they align with CFIR domains and constructs. Due to the considerable heterogeneity of how procedures of items, constructs, and domains are operationalized across disciplines and across researchers regarding measurement development and adaptation [33], we have chosen this three-focused coding approach. The CFIR’s 5 domains and 39 constructs are presented in Table 2.

### 2.8. Psychometric and Pragmatic Coding

We will apply commonly used criteria for psychometric and pragmatic coding, such as validity (face/content, construct, criterion), reliability (internal consistency, test–retest), the PAPERS scale [47], and measurement equivalence (invariance) as defined by Putnick and Bornstein [46]; the results are summarized in Table 3. The rating scale includes five pragmatic measurement characteristics that reflect the ease or difficulty of use. Nine psychometric measurement characteristics are included in the rating scale to assess reliability and validity. All properties of the PAPERS scale are rated on six levels with predefined values; poor (-1), none (0), minimal/emerging (1), adequate (2), good (3), or excellent (4). Additionally, we have chosen to include a tenth psychometric property—invariance—reflecting measurement equivalence. This is due to its importance as a prerequisite to comparing group means, and is most commonly tested through structural equation modelling using confirmatory factor analysis [46]. Invariance will be descriptively assessed, and not rated against any scale.

### 2.9. Analysis and Synthesis

The reporting of the results will be done through both narrative descriptions and descriptive statistics, using the proportions and frequencies of the psychometric and pragmatic properties, and CFIR domains and constructs.

## 3. Discussion

This systematic review protocol gives a detailed description for identifying measurements assessing factors that influence implementation in primary and secondary school settings. The current review’s systematic work will contribute to covering the fast-growing field of implementation science, expanding on knowledge from previous reviews. Psychometric evidence is key to producing confidence in the results obtained when measuring factors influencing implementation [47]. By identifying measurements that are psychometrically and pragmatically strong in the field, this review can contribute to the identification of feasible, effective, and sustainable implementation strategies. Through highlighting the gaps within the range of constructs in the tools retrieved, this review may provide insights for future research and resource allocation. The review includes measurements assessing key stakeholders embedded at multiple ecological levels within school settings themselves, and outer settings influencing the school context. Another strength of this systematic review protocol is that we include psychometric equivalence (invariance) as a more contemporary framework with structural equation modelling (SEM) [46], rather than limiting the scope to a more classical test-theory-informed measurement development, a mentioned limitation of the PAPERS scale [24]. Due to the fact that measurement invariance is a prerequisite to comparing group means, our systematic review will provide further understanding of the extent to which the included measurements have the ability to detect whether a construct has the same meaning across groups or across measurements over time.

A limitation of our review is that we exclude abstracts describing, e.g., dissertations and grey literature to detect measurements. Another limitation is that we limit our scope to factors influencing implementation, rather than also including ‘implementation outcomes’, as other earlier reviews have done in line with the procedures of the Instrument Review Project at SIRC [24]. In addition, we will not include empirical research and make ‘measurement packages’ through citation searches as elected by Lewis and colleagues [24]. Therefore, relevant empirical articles describing the further psychometric and pragmatic development of an original measurement may be missed. We address this limitation by including adapted measurements in our eligibility criteria, in addition to originally developed measurements. Even though the CFIR covers many domains and constructs, there might be relevant ones that will be missed due to the utilization of this specific framework. Lastly, only articles published in English and the research team’s native (Nordic) languages will be included, and therefore relevant articles in other languages may be missed. The decision to accept the abovementioned limitations is a result of pragmatic reasons, such as limited time and staff.

In sum, this review can provide a greater understanding of the factors influencing the implementation of innovations within educational settings and how these can be further studied. It offers insights into which quantitative measures are easy-to-use, reliable, and valid in the emerging field of implementation science.

## Figures and Tables

**Table 1:** Eligibility criteria.

No.	Criteria
i	Publications from peer-reviewed journal articles based on original research, in English and Nordic languages (Swedish, Norwegian, Danish, and Icelandic), and published from the year 2000
ii	Reported research from school settings (including primary and secondary school, excluding preschool, tertiary, and vocational education), involving school stakeholders’ such as students, teachers, school leaders and management, school nurses, psychologists, assistants, and educators or similar school staff
iii	Reported details concerning implementation measurement development or adaptation within educational-, behavioral-, and health studies broadly constructed, including both validated and non-validated measurements
iv	Reported psychometric and pragmatic properties
v	Measurements which assessed the content aligned with at least one of the Consolidated Framework for Implementation Research (CFIR) domains

**Table 2:** The Consolidated Framework of Implementation Research (CFIR) with its domains, constructs, and their descriptions*.

Domain	Construct	Description
Innovation Characteristics	Innovation Source	Whether key stakeholders perceive an intervention as internally or externally developed.
	Evidence Strength and Quality	How the quality and validity of evidence of an intervention are perceived by stakeholders.
	Relative Advantage	How the advantage of an intervention is perceived by stakeholders in relation to an alternative solution.
	Adaptability	The degree to which the core components of an intervention can be adapted and tailored towards local needs.
	Trialability	How an intervention can be tested on a small scale, and the reversibility of its implementation if warranted.
	Complexity	How difficult the implementation of an intervention is perceived to be by stakeholders. This is reflected by the duration, scope, radicalness, disruptiveness, centrality, and the intricacy and number of steps required to implement.
	Design Quality and Packaging	Stakeholders’ perception of how an intervention is presented.
	Cost	Costs connected to an intervention such as investments and supply, as well as the costs of the intervention itself.
Outer Setting	Patient Needs and Resources	How well-known and prioritized individual needs are within the organization, including the barriers and facilitators to meeting those needs.
	Cosmopolitanism	How an organization is networked with other (external) organizations.
	Peer Pressure	The pressure to implement an intervention for competitive or mimetic reasons among organizations.
	External Policy and Incentives	Includes a broad content of external strategies to disseminate interventions, along with policy, regulations, and guidelines, etc.
Inner Setting	Structural Characteristics	The architecture of an organization, involving size, maturity, age, etc.
	Networks and Communications	The nature and quality of formal and informal social networks and communications in an organization.
	Culture	An organization’s norms and values.
	Implementation Climate	An organization’s capacity and receptivity for change, along with the reward and support that is given for the use of a specific intervention. This construct contains six additional sub-constructs; tension for change, compatibility, relative priority, organizational incentives and rewards, goals and feedback, and learning climate.
	Readiness for Implementation	An organization’s commitment to the decision of the implementation of an intervention. This construct contains three additional sub-constructs; leadership engagement, available resources, and access to knowledge and information.
Characteristics of Individuals	Knowledge and Beliefs about the Intervention	The attitudes and values of individuals in connection to the intervention, as well as their familiarity with the content and principles of the intervention.
	Self-efficacy	How individuals perceive their own capabilities to execute the implementation.
	Individual Stage of Change	The characterization of the stage an individual is in, in relation to their use of the intervention.
	Individual Identification with Organization	How individuals perceive the organization, as well as their degree of commitment to it.
	Other Personal Attributes	A broad construct that involves other individual traits.
Process	Planning	The degree to which an intervention and its content for implementation is designed and developed in advance, as well as the quality of the content in that plan.
	Engaging	How individuals are involved in the implementation and use of the intervention. This construct contains four additional sub-constructs; opinion leaders, formally appointed internal implementation leaders, champions, and external change agents.
	Executing	How the implementation is actually carried out, in relation to the plan.
	Reflecting and Evaluation	Feedback about the progress and quality of an implementation, and reflections concerning experiences of the implementation.

* Based on the original work of Damschroder and colleagues [19,49].

**Table 3:** Psychometric and pragmatic domains and their definitions.

Psychometric and Pragmatic Properties	Domain	Definition
PAPERS Scale [24,47,48]		
Pragmatic Criteria	Length	Number of items
	Language	The readability of the items included in the measure
	Cost	The cost researchers pay to use the instrument
	Assessor Burden (Ease of training)	The required training needed for the assessor, and the administration of an instrument
	Assessor Burden (Ease of Interpretation)	The requirements to interpret the data from a measurement; the complexity of scoring interpretation
Psychometric properties criteria	Internal Consistency	Assesses reliability and indicates whether several items that measure the same construct produce similar scores (Cronbach’s α)
	Convergent Construct Validity	The degree to which constructs that are theoretically related are in fact related (e.g., effect size, Cohen’s *d*, or correlation, Pearson’s *r*)
	Discriminant Construct Validity	The degree to which constructs that are theoretically distinct are in fact distinct (e.g., effect size, Cohen’s *d*, or correlation, Pearson’s *r*)
	Known-Groups Validity	The extent to which the measure can differentiate groups known to have different characteristics
	Predictive Criterion Validity	The degree to which a measurement can predict or correlate with an outcome of interest measured at a future time (e.g., Pearson’s *r*)
	Concurrent Criterion Validity	Assesses whether measurements taken at the same time correlate, and if a measure’s observed scores correlate with scores from a previously established measure of the construct (e.g., Pearson’s *r*)
	Structural Validity	Known as the test structure, and refers to the degree to which a measure’s items increase or decrease together (e.g., assessed in nine ways*)
	Responsiveness	The ability to which a measure can detect clinically important changes over time (e.g., standardized response mean = SRM, Pearson’s *r*)
	Norms	Assesses generalizability based on the sample size, means, and standard deviations of item values
Measurement Equivalence [46]		
Psychometric Properties criteria	Invariance	Assesses the psychometric equivalence of a construct across groups or measurement occasions, and demonstrates that a construct has the same meaning across groups or across repeated measurements. Measurement invariance is a prerequisite to comparing group means, and is most commonly tested through structural equation modelling (SEM) using confirmatory factor analysis (CFA).
Content validity [45,50]		
Psychometric Properties criteria	Evaluation by Expert and Target Population	Evaluates each of the items constituting the domain for content relevance, representativeness, and technical quality by experts, and of actual experience from the target population
Reliability [45,50]		
Psychometric Properties criteria	Test–retest, Inter-rater, Intra-rater	Assesses to what degree a participant’s performance is repeatable, and how consistent their scores are across time

* Normed fit index = NFI; incremental fit index = IFI; goodness of fit index = GFI; Tucker–Lewis index = TLI; comparative Fit Index = CFI; relative non-centrality fit index = RNI; standardized RMR = SRMR; root mean square; error of approximation = RMSEA; weighted root mean residual = WRMR.

## Data Availability

Not applicable.

## Supplementary Materials

The following supporting information can be downloaded at: https://www.mdpi.com/article/10.3390/ijerph191912726/s1, Additional File 1: PRISMA-P 2015 Checklist.
